# Relationships between MRI fat distributions and sleep apnea and obesity hypoventilation syndrome in very obese patients

**DOI:** 10.1007/s11325-017-1599-x

**Published:** 2017-12-02

**Authors:** C. D. Turnbull, S. H. Wang, A. R. Manuel, B. T. Keenan, A. G. McIntyre, R. J. Schwab, J. R. Stradling

**Affiliations:** 1 NIHR Biomedical Research Centre, University of Oxford, Oxford, UK; 2 Oxford Centre for Respiratory Medicine, Oxford University Hospitals NHS Foundation Trust, Oxford, OX3 7LE UK; 3 Division of Sleep Medicine, University of Pennsylvania School of Medicine, Philadelphia, PA USA; 4 Sleep Centre, Aintree University Hospital NHS Foundation Trust, Liverpool, UK; 5 Department of Radiology, Oxford University Hospitals NHS Foundation Trust, Oxford, UK

**Keywords:** Obesity, Hypoventilation, Obstructive sleep apnea, Magnetic resonance imaging

## Abstract

**Purpose:**

Obesity is associated with both obstructive sleep apnea (OSA) and obesity hypoventilation. Differences in adipose tissue distribution are thought to underlie the development of both OSA and hypoventilation. We explored the relationships between the distribution of upper airway, neck, chest, abdominal and muscle fat in very obese individuals.

**Methods:**

We conducted a cross-sectional cohort study of individuals presenting to a tertiary sleep clinic or for assessment for bariatric surgery. Individuals underwent magnetic resonance (MR) imaging of their upper airway, neck, chest, abdomen and thighs; respiratory polygraphy; 1 week of autotitrating CPAP; and morning arterial blood gas to determine carbon dioxide partial pressure and base excess.

**Results:**

Fifty-three individuals were included, with mean age of 51.6 ± 8.4 years and mean BMI of 44.3 ± 7.9 kg/m^2^; there were 27 males (51%). Soft palate, tongue and lateral wall volumes were significantly associated with the AHI in univariable analyses (*p* < 0.001). Gender was a significant confounder in these associations. No significant associations were found between MRI measures of adiposity and hypoventilation.

**Conclusions:**

In very obese individuals, our results indicate that increased volumes of upper airway structures are associated with increased severity of OSA, as previously reported in less obese individuals. Increasingly large upper airway structures that reduce pharyngeal lumen size are likely to lead to OSA by increasing the collapsibility of the upper airway. However, we did not show any significant association between regional fat distribution and propensity for hypoventilation, in this population.

**Electronic supplementary material:**

The online version of this article (10.1007/s11325-017-1599-x) contains supplementary material, which is available to authorized users.

## Introduction

Obesity is associated with significant comorbidities, including obstructive sleep apnea (OSA) and obesity hypoventilation syndrome (OHS). These conditions coexist in only some obese individuals, whereas others develop only one of the complications or do not develop either. The individual distribution of adiposity may be crucial in determining which patients develop OHS, OSA or both.

Obesity may cause OSA by different mechanisms. Increased neck circumference may cause increased external loading pressure on the airway leading to OSA [1–3]. The minimal cross-sectional airway area has been shown to be more predictive of OSA than neck circumference [4]. This may be because the minimal cross-sectional airway area is determined by both the size of upper airway structures, which are associated with upper airway collapsibility [5], as well as external loading pressures exerted by larger neck circumference. The size and adiposity of upper airway structures have also been shown to be important and OSA patients have larger tongues with increased adiposity on volumetric magnetic resonance (MR) analysis [6]. Increased abdominal visceral adiposity decreases lung volumes, including the functional residual volume, which reduces traction on the pharynx and has been hypothesised to lead to increased pharyngeal collapsibility and, thus, OSA [7].

The apnea hypopnea index (AHI) is the most common measure of OSA severity. The AHI is influenced by the anatomic susceptibility of the upper airway to collapse along with dilator muscle responsiveness and other factors [8]. Autotitrating CPAP (autoCPAP), in addition to commonly being used in the treatment of OSA [9], delivers interesting information about upper airway function. AutoCPAP machines vary between models, but all increase the delivered pressure in response to residual obstructive events (snoring, hypopnea, apnea and flow limitation) [10, 11]. These events occur due to collapsing forces acting on the pharynx during sleep, and therefore, the therapeutic CPAP setting delivered can also be used as a measure of collapsing forces in the pharynx [12]. The autoCPAP setting is essentially at the opposite end of the pharyngeal compliance curve to P_CRIT_.

The underlying mechanisms of OHS are not fully understood, but regional variations in fat distribution are believed to play a role. Abdominal adiposity is known to be associated with decreased lung volumes and hypoventilation [13]. In addition, intrathoracic fat may pose a mechanical restriction on lung expansion, reducing lung volumes, and intramuscular adiposity (including diaphragmatic adiposity) has been postulated to reduce muscle strength, therefore leading to impaired lung expansion.

OHS diagnostic criteria include elevated daytime arterial carbon dioxide partial pressure (PaCO_2_), obesity, and no other aetiology present to explain the ventilatory failure. Arterial base excess represents renal compensation via increased bicarbonate levels in response to periods of hypercapnia. Our group has previously published data from this cohort showing that an intermediate group of patients, with an elevated bicarbonate level, but with a normal daytime PaCO_2_, is likely to represent early OHS [14].

In this manuscript, we explore the relationships between upper airway, thoracic, abdominal and intramuscular fat distributions, and OSA and hypoventilation. The aims of this analysis were to establish that the severity of OSA relates to measures of upper body obesity and the volumes of upper airway structures on MRI, and that the severity of hypoventilation relates to MRI measurements of visceral and upper body obesity in very obese individuals.

## Methods

This was a prospective observational study conducted in a single tertiary care hospital of individuals presenting to sleep clinic or for bariatric surgery. We have utilised unreported MR imaging (MRI) data from a previously published obesity and ventilatory failure trial [14]. Thus, this paper represents an exploratory, hypothesis-generating analysis. The study was registered prospectively with a global trials registry site (ClinicalTrials.gov, NCT01380418). The study was approved by local research ethics committee (Oxfordshire REC B 11/H0605/9) and conducted in accordance to the Declaration of Helsinki. All participants included in the study gave written informed consent.

Individuals underwent magnetic resonance (MR) imaging of their upper airway, neck, chest, abdomen and thighs; overnight in-lab polygraphy; 1 week of autotitrating CPAP; and early morning arterial blood gas to determine PaCO_2_ and base excess (BE). Individuals taking diuretics, theophylline, respiratory stimulants or respiratory depressants were excluded. Full details of the subjects included, and blood gas analysis can be found in the online supplement.

### MRI and soft tissue analysis

MRI of the upper airways, neck, chest and abdomen was performed. Details of MRI acquisition can be found in the online supplement.

All images were manually examined using Amira image analysis software (Amira 5.4.3. Visage Imaging, Inc., San Diego, CA). Upper airway structures were segmented individually between the posterior nasal spine and the level of the hyoid. This allows calculation of the volumes of different upper airway structures, as we have performed in other studies [6, 15, 16].

Using the Amira image software, volumes (cm^3^) were calculated of the following upper airway structures: the soft palate, the tongue, the parapharyngeal fat pads and the airway lateral walls. Neck and chest fat analysis extended from the inion to the C7-T1 intervertebral space and from the C7-T1 level to the diaphragm, respectively. The abdominal fat analysis used the L2–L3 and L3–L4 landmark slice visceral fat measurements, as these have been shown to be predictive of total visceral adipose tissue [17].

Thigh fat measurement was conducted by first defining a border along the fascia lata and then measuring the fat within the fascial compartment, as well as within the muscles from the neck of the femur to the superior aspect of the patella. The ratio of the intrafascial fat divided by the intrafascial muscle was calculated as a measure of intramuscular adiposity.

### Cardiorespiratory polygraphy

Baseline one-night, level 3, in-hospital cardiorespiratory polygraphy (Win-Visi monitoring system; Stowood Scientific Instruments, Oxford, UK) was performed. This included nasal pressure via nasal cannula, oximetry, pulse rate and respiratory effort via thoracic and abdominal bands. Body movements (derived automatically from the video signal), heart rate and pulse transit time changes were also routinely recorded as markers of arousal from sleep. Apnea was defined as ≥ 10 s with < 10% airflow, and hypopnea as ≥ 10 s with 50% reduction in nasal airflow (or summed thoraco-abdominal movement in the absence of a nasal flow signal) with > 4% oxygen desaturation. The apnea hypopnea index (AHI) was defined as the number of apneas and hypopneas per hour of sleep. ODI was calculated as the number of oxygen desaturations > 4% per hour of sleep.

### AutoCPAP

Regardless of AHI, all patients tried autoCPAP (S9 Elite, ResMed, Abingdon, UK). Only individuals using CPAP for at least seven nights and with an average usage of greater than 4 h per night had CPAP their 95th centile autoCPAP setting recorded.

### Statistical analysis

Detailed statistical methods can be found in the online supplement. Subjects were divided into categories by the presence/absence of OSA (AHI > 15) and the presence/absence of a raised base excess (≥ 2.0 mmol/l). Differences between these categories were compared using unpaired *t* tests.

The relationships between sleep parameters (autoCPAP and AHI) and MRI variables were explored using univariable linear regression. Log and square root transformations were performed where necessary. Similarly, the relationships between hypoventilation measures (BE and PaCO_2_) and MRI variables were explored using univariable linear regression.

The relationships between potential confounding variables (age, gender and height) and MRI variables, OSA measurements and hypoventilation measurements were explored using univariable and multivariable linear regression. As a further analysis, multivariable associations between MR imaging variables and AHI/autoCPAP/BE/PCO_2_ were analysed for any significant univariable associations adjusting for significant confounders (*p* < 0.10).

As this is considered an exploratory, hypothesis-generating analysis, no correction for multiple comparisons or sample size/power analysis has been performed.

## Results

Seventy-two patients were recruited to the study, of whom 53 (74%) underwent MR imaging of the upper airway; thus, only these 53 patients were included in this analysis. Baseline characteristics are shown in Table 1. Nineteen percent of patients met criteria for mild OSA (AHI 5–15, 9 of 48), 8% met criteria for moderate OSA (AHI 15–30, 4 of 48) and 40% met criteria for severe OSA (AHI > 30, 19 of 48). Eighteen percent (9 of 51) met criteria for OHS (PaCO_2_ > 6.0 kPa) and 53% (27 of 51) met our previously described criteria for early OHS (BE ≥ 2 mmol/l) [14].

**Table 1:** Baseline characteristics for all subjects

	*N*	Mean, median, or number	SD, IQR, or percentage
Age (years)	53	51.6	8.4
Gender: number male (%)	53	27	50.9%
BMI (kg/m^2^)	53	44.3	7.9
Neck circumference (cm)	53	45.9	5.1
Waist circumference (cm)	52	129.5	12.2
ESS	43	13.1	5.7
AHI (/hour)	48	12.9	4.3, 56.7
ODI (/hour)	48	45.9	15.1, 70.1
AutoCPAP setting (cmH_2_O)	42	12.1	2.6
BE (mmol/l)	51	1.9	2.2
PaCO_2_ (kPa)	51	5.4	0.53

All 53 patients had satisfactory upper airway MR imaging, 50 had satisfactory neck and chest MR imaging and 52 had satisfactory thigh muscle MR imaging. Only 31 (59%) patients had satisfactory abdominal MR imaging, with 15 scans not containing the area of interest, 5 affected by motion artefact and 2 being unable to appropriately landmark. MRI variable measurements are shown in Table 2 for all patients, along with comparisons divided by the presence/absence of OSA and the presence/absence of a raised base excess.

**Table 2:** Mean MRI variable values for all patients and divided by the presence/absence of OSA (AHI > 15) and divided by the presence/absence of a raised base excess ≥ 2.0 mmol/l. Results are shown as mean ± standard deviation. Differences between groups were compared using an unpaired *t* test

	MRI variable	All patients	OSA category				BE category			
			AHI ≤ 15	AHI > 15	Difference in means (95% CI)	*p*	BE < 2.0 mmol/l	BE ≥ 2.0 mmol/l	Difference in means (95% CI)	*p*
Upper airway	Soft palate (cm^3^)	9.8 ± 3.2	8.1 ± 2.7	11.5 ± 2.8	+ 3.4 (+ 1.8 to + 5.0)	*< 0.001*	10.3 ± 2.5	9.6 ± 3.7	− 0.6 (− 2.4 to + 1.1)	0.48
	Tongue (cm^3^)	124.1 ± 26.1	108.3 ± 17.5	140.0 ± 23.0	+ 31.7 (+ 19.9 to + 43.5)	*< 0.001*	125.1 ± 20.9	125.1 ± 30.4	− 0.0 (− 14.9 to + 14.8)	0.99
	Lateral walls (cm^3^)	24.8 ± 8.5	20.3 ± 5.7	29.0 ± 9.1	+ 8.6 (+ 4.1 to + 13.1)	*< 0.001*	24.2 ± 7.2	26.0 ± 9.5	+ 1.8 (− 3.0 to 6.6)	0.45
	Parapharyngeal fat pads (cm^3^)	3.3 ± 1.4	3.1 ± 1.3	3.3 ± 1.5	+ 0.2 (− 0.7 to + 1.0)	0.71	3.5 ± 1.3	3.3 ± 1.6	− 0.2 (− 1.0 to + 0.6)	0.55
Neck	Neck visceral fat (cm^3^)	412.8 ± 174.4	342.0 ± 139.0	481.3 ± 181.0	+ 139.2 (+ 41.9 to + 236.6)	*0.006*	392.3 ± 159.8	440.3 ± 183.4	+ 48.0 (− 51.4 to + 147.5)	0.34
	Submental fat (cm^3^)	79.8 ± 30.2	76.6 ± 31.6	81.8 ± 30.6	+ 5.2 (− 13.5 to + 23.9)	0.58	76.9 ± 30.9	83.8 ± 29.7	+ 6.9 (− 10.5 to + 24.3)	0.43
Chest	Chest visceral fat (cm^3^)	1783.6 ± 757.1	1585.4 ± 648.9	1899.1 ± 726.6	+ 313.8 (− 101.1 to + 728.6)	0.14	1638.0 ± 548.2	1945.5 ± 884.5	+ 307.5 (− 122.2 to + 737.2)	0.16
Abdomen	L2–L3 Visceral fat (cm^2^)	298.9 ± 120.5	231.0 ± 69.7	361.5 ± 133.6	+ 130.5 (+ 46.9 to + 214.1)	*0.003*	315.9 ± 124.7	289.8 ± 118.9	− 26.2 (− 117.3 to + 65.0)	0.56
	L3–L4 Visceral fat (cm^2^)	297.0 ± 108.2	245.9 ± 83.9	343.5 ± 114.4	+ 97.6 (+ 21.5 to + 173.6)	*0.01*	312.0 ± 107.2	288.0 ± 112.7	− 23.9 (− 106.2 to + 58.3)	0.56
Thigh	Intramuscular fat/muscle ratio	0.34 ± 0.13	0.36 ± 0.15	0.30 ± 0.09	− 0.06 (− 0.13 to + 0.01)	0.09	0.32 ± 0.12	0.35 ± 0.13	0.03 (− 0.04 to 0.11)	0.33

p < 0.05 are highlighted in italics

### Univariable linear regression analyses

AHI was not normally distributed, and therefore, the AHI was square root transformed to give an approximately normal distribution. The univariable associations between MR imaging variables and OSA measurements (square root of the AHI and AutoCPAP setting) are displayed in Table 3. There were significant associations between the volume of the soft palate, tongue, lateral walls, neck visceral fat, and L2–L3 visceral fat area, and the AHI; and between the soft palate volume and the AutoCPAP setting.

**Table 3:** Univariable associations between both the AHI and AutoCPAP pressure and MR imaging variables. AHI and autoCPAP setting were the dependent variables with MRI variables were the independent variables. Beta coefficients are standardised (e.g. a change in standardised beta of 0.5 means that 1 one SD change in the independent variable would lead to a 1.0 SD change in the dependent variable)

		Square root transformed AHI		AutoCPAP setting (cmH_2_O)	
		Beta	*p*	Beta	*p*
Upper airway	Soft palate (/cm^3^)	*0.54*	*< 0.001*	*0.37*	*0.02*
	Tongue (/cm^3^)	*0.55*	*< 0.001*	0.29	0.06
	Lateral walls (/cm^3^)	*0.49*	*< 0.001*	0.26	0.10
	Parapharyngeal fat pads (/cm^3^)	0.08	0.58	0.05	0.74
Neck	Neck visceral fat (/cm^3^)	*0.44*	*0.003*	0.20	0.22
	Submental fat (/cm^3^)	− 0.08	0.62	0.07	0.65
Abdomen	L2–L3 Visceral fat (/cm^2^)	*0.42*	*0.02*	0.34	0.12
	L3–L4 Visceral fat (/cm^2^)	0.34	0.08	0.34	0.13
Thigh	Intramuscular fat/muscle ratio	− 0.28	0.10	− 0.28	0.07

p < 0.05 are highlighted in italics

The univariable associations between MR imaging variables and hypoventilation measurements are displayed in Table 4. There were no significant associations; increased chest visceral fat showed a borderline (*p* = 0.05) association with higher BE.

**Table 4:** Univariable associations between both PaCO2 and BE, and MR imaging variables. PaCO2 and BE were the dependent variables with MRI variables were the independent variables. Reported beta coefficients are standardised (e.g. a change in standardised beta of 0.5 means that 1 one SD change in the independent variable would lead to a 1.0 SD change in the dependent variable)

		PaCO_2_ (kPa)		Base excess (mmol/l)	
		Beta	*p*	Beta	*p*
Neck	Neck visceral fat (/100 cm^3^)	0.13	0.37	0.08	0.60
	Submental fat (/100 cm^3^)	0.14	0.35	0.16	0.27
Chest	Chest visceral fat (/100 cm^3^)	0.12	0.43	0.28	0.05
Abdomen	L2–L3 Visceral fat (/100 cm^2^)	0.29	0.13	0.22	0.25
	L3–L4 Visceral fat (/100 cm^2^)	0.23	0.23	0.21	0.27
Thigh	Intramuscular fat/muscle ratio	0.03	0.84	0.13	0.37

The univariable and multivariable associations between the potential confounders of age, gender and height, and the MRI variables, OSA measures and hypoventilation measures were assessed and the results are shown in the online supplement e-Table 1 and *e-*Table 2, respectively.

After adjusting for other potential confounders in multivariable analysis, male gender was associated with increased volumes of the soft palate (*p* = 0.004), tongue volume (*p* < 0.001), and lateral walls (*p* < 0.001) and a decreased intramuscular fat/muscle ratio (*p* = 0.003); taller height was associated with increased neck (*p* = 0.01) and chest (p = 0.01) visceral fat volumes; both male gender (*p* = 0.02) and taller height (*p* = 0.001) were associated with increased submental fat volumes; there were trends towards significant associations between both age (*p* = 0.08) and height (*p* = 0.09) with L2–L3 visceral fat; there was a trend towards a significant association between age (*p* = 0.05) and L3–L4 visceral fat.

### Multivariable linear regression

Multivariable linear regression analyses were performed for variables with *p* < 0.10 in univariable linear regression analyses. Potential confounders were included as covariates where *p* < 0.10.

There remained a significant association between increasing volume of the soft palate and increasing square root transformed AHI (standardised beta = 0.33, *p* = 0.04), with gender as a covariate. There was a trend towards increasing volume of the tongue and increasing square root transformed AHI (standardised beta = 0.33, *p* = 0.06), with gender as a covariate. There was no significant association between the volume of the lateral walls (standardised beta = 0.18, *p* = 0.35) and the square root transformed AHI, with gender as a covariate. There were no significant associations between the neck visceral fat volumes (standardised beta = 0.19, *p* = 0.26) or L2–L3 visceral fat area (standardised beta = 0.06, *p* = 0.79) and the square root transformed AHI, with height as a covariate.

There was a trend towards increasing volumes of the soft palate being associated with increasing autoCPAP settings (standardised beta = 0.33, *p* = 0.08), with gender as a covariate. There was no association between the volume of the tongue (standardised beta = 0.21, *p* = 0.37) or the intramuscular fat:muscle ratio (standardised beta = − 0.20, *p* = 0.26) and the autoCPAP setting, with gender as a covariate.

There was a trend towards increasing chest visceral fat volumes being associated with an increased base excess (unstandardized beta = 0.28, *p* = 0.07), with height as a covariate.

## Discussion

We describe the relationships between increasing volumes of several upper airway structures and both increasing AHI and autoCPAP setting in a population of very obese individuals. Increased volumes of the soft palate and tongue were associated with increased AHI and the autoCPAP setting. Gender was a significant covariate in these analyses. Increased volume of the soft palate remained a significant predictor of increased AHI after allowing for gender. In our cohort of very obese individuals, we found no particular pattern of fat distribution associated with hypoventilation, although there was a trend towards an association between chest visceral fat and an increased base excess. These outcomes are post hoc analyses from a cross-sectional observation data and as such are considered exploratory and hypothesis-generating only.

### Obesity and OSA

An anatomical susceptibility of the upper airway to collapse is associated with an elevated AHI [8]. Although the AHI increases with increasing upper airway collapsibility, it is also dependent on other factors, such as upper airway dilator muscle responsiveness and other factors [8, 18]. Our data suggest that in very obese individuals, increased tongue and soft palate volumes are associated with increased severity of obstructive sleep apnea, likely due to an increased propensity of the upper airway to collapse. Indeed, the mean tongue volume was large in this cohort both for those with OSA (140 ± 23.0 cm^3^) and those without OSA (108.3 ± 17.5 cm^3^), when compared with previous studies examining both OSA patients (101.2 ± 17.7 cm^3^) and controls (85.5 ± 13.8 cm^3^) [6]. The finding that the increased volumes of the soft palate and tongue were associated with the AHI but not neck fat raises the hypothesis that intraluminal narrowing of the upper airway by soft tissue structures is more important that extraluminal loading of the upper airway in determining OSA risk in very obese individuals.

AutoCPAP was considered as a potential alternative to the critical closing pressure of the pharynx (P_CRIT_) as a measure of the collapsibility of the upper airway. P_CRIT_ is concerned with the pharynx at its point of collapse, and the autoCPAP setting describes the pressure needed to hold open the airway to the point of minimal flow limitation, making it a reasonable proxy measure [12]. Contrary to our expectations, whilst the volumes of upper airway structures were related to the AHI, only the soft palate volume and not the tongue had a trend towards association with the autoCPAP setting in multivariable analyses. There are several possible reasons why larger soft palate and tongue volumes were not associated with increased autoCPAP settings. There is variable genioglossus muscle activity on CPAP [19, 20], variable genioglossus activation in obese individuals in response to airway obstruction [18], a loss of the relationship between neck circumference and CPAP pressure in this very obese population [21] and flow limitation even in those without OSA [22], which autoCPAP may respond to.

### Obesity and hypoventilation

We did not show associations between MR imaging measures of visceral adiposity or of intramuscular fat with measures of hypoventilation. There was a trend towards increased chest visceral adiposity and increased base excess. This is in contrast to our previous finding in this cohort that dual-energy X-ray absorptiometry (DEXA) assessments of abdominal visceral adiposity were correlated with BE [23]. It was only possible in this current study to estimate abdominal visceral adipose tissue on single MRI slices in a subset of patients, which limits the conclusions that can be drawn. Furthermore, the DEXA measurements reported previously were technically possible only in patients with a BMI < 40 kg/m^2^ and not those with a higher BMI, many of whom were included in this MRI analysis. Thus, the two populations in the two studies are different. It is possible that the relationship between visceral adiposity and propensity to hypoventilation reaches a plateau above a BMI of 40 kg/m^2^, and that beyond a certain threshold other factors, such as impaired ventilatory responses, are required for hypercapnia to occur.

### Limitations

The sample size is an important limitation of this study, particularly for abdominal MR imaging, where only 31 patients had adequate imaging. It is possible that a modest association between abdominal visceral adiposity and hypoventilation may have been missed. A limitation of this current study is that only a small proportion of patients met criteria for established hypoventilation (18%), though 27 (53%) had a raised BE. In this study, no patients had a daytime PaCO_2_ > 7 kPa, and therefore, we cannot make conclusions about the relationship between MRI measurements of adiposity and more severe hypoventilation. In addition patients included were all obese and as a result had significant abdominal and thoracic adiposity. Therefore, there may be a relationship between visceral adiposity and hypoventilation over wider range obesity. However, this study does raise the hypothesis that over a certain threshold of visceral obesity other non-anatomical factors, such as physiological ventilatory responses, may determine the risk of hypoventilation.

The AHI, which was derived by polygraphy, and the autoset CPAP setting were not adjusted for sleep stage or position. As full polysomnography was not recorded, the AHI was not recorded by sleep stage or sleep position. As the autoset CPAP setting was derived from 1 week of therapy, it is unlikely to have been influenced by sleep stage. Therefore, it is possible that the time in different sleep stages or positions varied between individuals and may have biased the relationship between MRI measurements and the AHI or the autoset CPAP setting.

MR images were recorded during wakefulness. Rostro-caudal fluid shifts that occur at night are likely to further increase the volume of the upper airway soft tissue and further reduce cross-sectional area. However, as patients with congestive cardiac failure and other significant comorbidities were excluded, it is unlikely that rostro-caudal fluid shifts will have significantly influenced our results.

### Confounding variables

We found significant confounding relationships between gender and the volume of soft palate, tongue, lateral upper airway walls, neck visceral fat, estimates of abdominal visceral fat and the thigh intramuscular fat/muscle ratio.

Gender differences in the anatomy of the upper airway may well be an important determinant of the differences in collapsibility of the male and female upper airway. These results could be due to smaller tongue and soft palate sizes, as we have shown that the volume of these structures is associated with sleep apnea severity. Others have shown that females have smaller tongues, soft palate size and shorter airway length, and this was associated with a less collapsible upper airway [24]. Airway length may be a crucial factor, as shorter pharyngeal length is thought to reduce susceptibility to OSA.

Differing cut-offs in measurements of visceral adiposity for predicting the metabolic syndrome have been described in the literature for pre-menopausal women compared to post-menopausal women and men [25]. Most studies examining waist circumference as a predictor of hypercapnia in OSA have not assessed gender differences [26, 27]. The effect of gender on the relationship between abdominal visceral adiposity and hypoventilation warrants further investigation.

We found significant associations between height, gender and MRI measurements of adiposity. Male gender and not taller height mediated increasing volumes of upper airway structures, whereas taller height and not male gender mediated increased chest and neck fat volumes. The relationship between height and volume of major visceral adipose tissue is well established, as it is with the common measurement of BMI [28], where weight is divided by height squared. It seems that height is more important in determining visceral adipose tissue, but not adiposity in the upper airways structures.

Visceral abdominal tissue is known to increase with age and this has been linked with the metabolic syndrome [29]. Hormonal changes with ageing have been linked with increasing visceral adiposity, and changes around menopause [30], may account for interactions that we observed between age, gender and visceral abdominal fat.

## Conclusions

In these very obese individuals, we have shown that increased tongue and soft palate volumes are associated with increased severity of OSA. Increasingly large upper airway structures are likely to lead to OSA by increasing the collapsibility of the upper airway. Many associations were confounded by covariates of age, gender and height. Contrary to findings relating to measurements of OSA, we did not show any association between regional fat distribution and propensity for hypoventilation in this population. This contrasts with our previous finding in a different subset of very obese individuals, where visceral adiposity measured on DEXA scanning was predictive of hypoventilation in individuals with a BMI < 40 kg/m^2^. This disparity warrants further investigation into the role of abdominal fat in the genesis of obesity hypoventilation syndrome.

## Electronic supplementary material

ESM 1 (DOCX 24 kb)
