# Supplementary material for: Relationships between MRI fat distributions and sleep apnea and obesity hypoventilation syndrome in very obese patients
Source: Sleep Breath. 2017 Dec 2;22(3):673–81. doi: 10.1007/s11325-017-1599-x (PMC6133118; doi:10.1007/s11325-017-1599-x)
Supplement: Supplementary file 1 — (DOCX 24 kb) [file 11325_2017_1599_MOESM1_ESM.docx]

**Supplemental Material**

Relationships Between MRI Fat Distributions and Sleep Apnea and Obesity Hypoventilation Syndrome in Very Obese Patients

C. D. Turnbull *BMBCh*^1,2^, S. H. Wang *BA* ^3^, A. R. Manuel *DPhil*^4^, B.T. Keenan *MS*^3^, A. G. McIntyre *BAppSc*^5^, R.J. Schwab *MD* ^3^, J. R. Stradling *MD*^1,2^

**Methods**

Subjects

Patients were enrolled following referral to the sleep and ventilation clinic or during assessment for possible bariatric surgery, at a single tertiary centre (Oxford University Hospitals NHS Foundation Trust). Referrals were all comers and were not biased towards those with sleep symptoms. Detailed baseline clinical assessment included body mass index (BMI), height, weight, waist and neck circumference measurements, and Epworth Sleepiness Score (ESS).

Exclusion criteria included: the presence of obstructive lung disease (FEV_1_/FVC <70% predicted), other serious comorbidities, such as congestive cardiac failure, primary CNS or neuromuscular diseases, untreated hypothyroidism, or current treatment of OSA/OHS including CPAP or non-invasive ventilation.

All patients were invited to undergo magnetic resonance (MR) imaging, overnight in-hospital cardiorespiratory polysomnography and one-week of autotitrating CPAP. Only patients who underwent MR imaging are reported here.

MR imaging acquisition

Upper airway imaging was performed on the patients in the supine position during wakefulness. Standard T1 spin echo (SE) scans (5mm slice thickness) were performed. Chest imaging was performed in the supine position with sagittal T1 Fast Spin Echo (FSE) scans acquired (10mm slice thickness). Abdominal imaging was performed in the supine position using axial T1 FSE scans (10mm slice thickness). Axial T1 SE images of the thighs (10mm slice thickness) were acquired with subjects supine and the legs extended.

Blood gas analysis

Arterial blood gas sampling was performed from the radial artery with subjects in a seated position breathing room air between 8 and 10am after at least 15 minutes of rest. Analysis of PaO_2_, PaCO_2_, arterial standard (HCO_3_^-^), base excess (BE), and pH were performed using an analyser maintained and calibrated according to the manufacturer’s recommendations (ABL 90; Radiometer Medical ApS).

Statistical analysis

Statistical analysis was performed using SPSS^TM^ (version 20, IBM Corporation Ltd, USA). Normality was assessed for continuous variables and data are expressed as mean ± standard deviation (SD) where normally distributed or median (first quartile, third quartile) where non-normally distributed. Categorical variables are expressed as number (percentage).

The relationships between sleep parameters (autoCPAP setting and AHI) and MRI variables were explored using linear regression. MRI variables included: upper airway structures likely to directly narrow the upper airway lumen (volumes of the soft palate, tongue, lateral walls and parapharyngeal fat pads); structures likely to exert external loading pressure on the upper airway (neck visceral fat volume and submental fat volume); abdominal visceral fat likely to reduce functional residual capacity and increased pharyngeal collapsibility by reduced traction (L2-L3 visceral fat volume, L3-L4 visceral fat volume); and a measure of muscle fat infiltration (the thigh intramuscular fat/muscle ratio).

The relationships between hypoventilation measures (BE and PaCO_2_), and MRI variables were similarly explored using linear regression. MRI variables of interest included measures of visceral fat as markers of a distribution potentially related to mechanical restriction of lung volumes (neck visceral fat volume, submental fat volume, chest visceral fat volume, L2-L3 visceral fat volume, L3-L4 visceral fat volume) and a measure of muscle fat infiltration (thigh intramuscular fat/muscle ratio).

The relationships between confounding variables, MRI variables, OSA measurements and hypoventilation measurements were explored using univariable and multivariable linear regression.

**Results**

Confounding variables

Univariable associations between the potential confounders of age, gender and height, and the MRI variables, OSA measures, and hypoventilation measures were assessed and the results are shown in e-*Table 1*. There was a significant increase in abdominal visceral fat measurements with older age. Male gender was associated with increased volumes of the soft palate, tongue, lateral walls, neck visceral fat, abdominal visceral fat and a reduced intramuscular fat/muscle ratio. Taller height was associated with increasing volumes of the soft palate, tongue, lateral walls, neck visceral fat, submental fat, chest visceral fat and abdominal visceral fat. Male gender was associated with an increased AHI and PaCO_2_, and taller height was associated with increased AHI.

|  | | Age (/year) | | Male Gender | | Height (/cm) | |
| --- | --- | --- | --- | --- | --- | --- | --- |
|  |  | Beta | P | Beta | P | Beta | P |
| Upper airway | Soft Palate (cm^3^) | 0.072 | 0.18 | **4.2** | **<0.001** | **0.21** | **<0.001** |
|  | Tongue (cm^3^) | 0.35 | 0.43 | **37.9** | **<0.001** | **1.8** | **<0.001** |
|  | Lateral walls (cm^3^) | 0.056 | 0.70 | **12.8** | **<0.001** | **0.57** | **<0.001** |
|  | Parapharyngeal fat pads (cm^3^) | 0.026 | 0.28 | 0.41 | 0.30 | 0.033 | 0.13 |
| Neck | Neck visceral fat (cm^3^) | 3.5 | 0.24 | **205.2** | **<0.001** | **12.2** | **<0.001** |
|  | Submental fat (cm^3^) | 0.28 | 0.59 | 0.91 | 0.92 | **1.0** | **0.03** |
| Chest | Chest visceral fat (cm^3^) | 14.7 | 0.25 | 217.6 | 0.32 | **28.3** | **0.02** |
| Abdomen | L2-L3 Visceral fat (cm^2^) | **5.6** | **0.02** | **146.4** | **<0.001** | **8.0** | **<0.001** |
|  | L3-L4 Visceral fat (cm^2^) | **5.2** | **0.01** | **102.7** | **0.006** | **5.7** | **0.005** |
| Thigh | Intramuscular fat/muscle ratio | 0.001 | 0.58 | **-0.10** | **0.003** | -0.002 | 0.26 |
| OSA | Square root AHI | -0.02 | 0.73 | **3.1** | **<0.001** | **0.17** | **<0.001** |
|  | AutoCPAP (cmH_2_O) | 0.046 | 0.37 | 1.4 | 0.09 | 0.056 | 0.21 |
| Hypo-ventilation | BE (mmol/l) | 0.000 | 0.99 | 0.55 | 0.38 | 0.028 | 0.42 |
|  | PaCO_2_ (kPa) | 0.004 | 0.65 | **0.32** | **0.03** | 0.010 | 0.22 |

***e-Table 1:*** *Univariable associations between potential confounding variables, MR imaging variables, OSA variables and hypoventilations variables. MRI variables, OSA measures, or hypoventilation measures were the dependent variables and age, gender or height, were the independent variables. Reported beta coefficients are unstandardized.*

In addition to univariable associations, age, gender and height were entered into multivariable linear regression models to predict MRI variables (when p<0.10) and results are shown in *e-Table 2*. Gender, but not height, was an independent predictor of soft palate volume, tongue volume, and lateral walls volume. Height, but not gender, was an independent predictor of neck visceral fat, and chest visceral fat. Both male gender and height remained significant predictors of submental fat volumes. There were trends towards significant associations between both age and height with L2-L3 visceral fat, but not with gender. There was a trend towards a significant association between age and L3-L4 visceral fat, but neither gender nor height was associated.

|  | | Age | | Gender | | Height | |
| --- | --- | --- | --- | --- | --- | --- | --- |
|  |  | Beta | P | Beta | P | Beta | P |
| Upper airway | Soft Palate | - | - | **3.1** | **0.004** | 0.077 | 0.18 |
|  | Tongue | - | - | **31.5** | **<0.001** | 0.45 | 0.29 |
|  | Lateral walls | - | - | **12.0** | **<0.001** | 0.058 | 0.66 |
| Neck | Neck visceral fat | - | - | 90.7 | 0.12 | **8.4** | **0.01** |
|  | Submental fat | - | - | **30.1** | **0.02** | **2.3** | **0.001** |
| Chest | Chest visceral fat | - | - | 386.2 | 0.22 | **44.5** | **0.01** |
| Abdomen | L2-L3 Visceral fat | 3.6 | 0.08 | 38.0 | 0.54 | 5.5 | 0.09 |
|  | L3-L4 Visceral fat | 4.0 | 0.05 | 9.3 | 0.88 | 4.4 | 0.18 |
| Thigh | Intramuscular fat/ muscle ratio | - | - | **-0.10** | **0.003** | - | - |

***e-Table 2:*** *Multivariable linear regression model results for potential confounders age, gender and height with MRI variables. MRI variables were the dependent variables and age, gender and height were the independent variables. Multiple linear regression was performed by entering dependent variables with a p<0.10 in univariable analysis. – represent independent variables that were not significant in univariable analysis. Reported beta coefficients are unstandardized.*
